# Delta‐9 desaturase reduction in gastrointestinal cells induced to senescence by doxorubicin

**DOI:** 10.1002/2211-5463.13945

**Published:** 2024-12-10

**Authors:** Valentina De Nunzio, Emanuela Aloisio Caruso, Matteo Centonze, Giuliano Pinto, Miriam Cofano, Ilenia Saponara, Maria Notarnicola

**Affiliations:** ^1^ Laboratory of Nutritional Biochemistry National Institute of Gastroenterology IRCCS “Saverio de Bellis” Castellana Grotte Italy

**Keywords:** delta‐9 desaturase, gastrointestinal cells, lipidomic analysis, senescence

## Abstract

The condition of cellular senescence has specific features, including an altered lipid metabolism. Delta‐9 desaturase (Δ9) catalyzes the conversion of saturated fatty acids, such as palmitic acid and stearic acid, into their monounsaturated forms, palmitoleic and oleic acid, respectively. Δ9 activity is important for most lipid functions, such as membrane fluidity, lipoprotein metabolism and energy storage. The present study aimed to investigate differences in the expression of Δ9 in senescence‐induced pancreatic (MIA‐PaCa‐2 and PANC‐1) and hepatic (Hepa‐RG and HLF) cancer cell lines. Cellular senescence was induced by growing cells in the presence of the chemotherapic drug doxorubicin. Senescence status was determined by the senescence‐associated beta‐galactosidase activity assay kit combined with the p21 and senescence associated secretory phenotype protein assay. Δ9 was downregulated in all senescence‐induced cell lines compared to control cells, in both the lipidomic analysis and when measuring protein levels via western blotting. Hence, our findings demonstrate that the study of membrane lipid composition and the expression levels of Δ9 could potentially form the basis for future applications investigating the state of cellular senescence.

## Graphical abstract

Pancreatic and hepatic cell lines induced to senescence by doxorubicin treatment were used for lipidomic analysis. A downregulation of Δ9 and a reduced MUFA/SFA ratio was found in all senescence‐induced cells compared to untreated control cells.

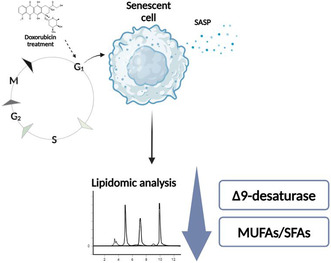

**Abbreviations**
Δ9: delta‐9 desaturase
CTR: control cells
DOXO: doxorubicin
FA: fatty acid
FAME: fatty acid methyl ester
FASN: fatty acid synthase
GC: gas chromatography
IL‐6: interleukin‐6
MUFA: monounsaturated fatty acid
PAI‐1: plasminogen activator inhibitor‐1
PUFA: polyunsaturated fatty acid
RB: retinoblastoma protein
SASP: senescence associated secretory phenotype
SA‐β‐gal: senescence‐associated beta‐galactosidase
SCD1: stearoyl‐CoA desaturase‐1
SFA: saturated fatty acid

Cell senescence is a state of irreversible growth arrest induced in response to intrinsic and extrinsic stimuli such as telomere shortening, radiation, DNA damage, oxidative and genotoxic stress. A senescent cell is characterized by a specific phenotype with changes in cellular morphology, metabolic profile and chromatin structure, associated with the expression of cell cycle inhibitors [1, 2].

In tissues, the chronic accumulation of senescent cells contributes to the decline in the health of the organism and the onset of several age‐related diseases [3]. Recent evidence suggests that the age‐associated increase in the number of senescent cells contributes to the decline in immune function [4], as well as to an alteration of cell apoptotic regulation [5]. In cellular senescence and aging, a dysregulation of expression of senescence‐related genes, such as p53 and p21, has been observed [6]. p53 is classified as an oncosoppressor and it has been found to be mutated in 50–55% of human cancers. p53 gene mutations hinder the the ability of p53 protein to facilitate DNA repair in response to damage. In tumors, p53 can be inactivated by other mechanisms, such as amplification of oncogenes or translocation into the cytoplasm. p53 regulates the G1/S phase of the cell cycle by promoting the accumulation of p21, an inhibitor of the CDK4/cyclinD1 complex. Inactivation of the CDK4/cyclinD1 complex allows retinoblastoma proteins (RBs) to block E2F and consequently arrest the cell cycle. In the absence of p53, the arrest of cell cycle does not occur, and therefore cells proliferate [7].

Furthermore, positive regulation of p53/p16 induces senescence. Senescent cells show an irreversible cell cycle arrest. The main molecular regulators of this irreversible process appear to be p53–p21CIP1 and p16INK4a–RB [2, 8]. The accumulation of p21 and p16 leads to the activation of RB family proteins which, in cascade, inhibit the activation of the E2F factor and consequently cell cycle arrest occurs.

Senescent cells can also influence neighboring cells in a paracrine manner through a complex secretome of cytokines and other signaling molecules, increasing inflammation and immune cell activation [9]. This phenomenon is known as the senescent associated secretory phenotype (SASP) and is a specific feature for senescent cells [10].

Several studies have demonstrated that cellular senescence is also correlated to an altered lipid metabolism [11, 12].

Lipids are biomolecules with multiple functions that are useful for cell growth and maintenance and they play a direct role in the structure and energetics of the cell. Most lipids can be classified into fatty acids (FAs) and their derivatives, such as triglycerides and glycerophospholipids, and in sphingolipids and sterols, such as cholesterol. FAs are required in cells for energy storage, membrane function and the generation of signaling molecules, at the same time as serving as an important source of energy through mitochondrial‐mediated beta‐oxidation [13]. In addition, their content may be a useful biomarker to assess the state of cell membranes and understand the role of lipids in disease, inflammation and senescence [13, 14, 15].

Based on their structure, FAs can be divided in two main groups: saturated FAs (SFAs), which do not contain any double bonds in their carbon chain, and unsaturated FAs. The latter, depending the degree of unsaturation, can be distinguished into monounsaturated fatty acids (MUFAs) or polyunsaturated fatty acids (PUFAs). The degree of fatty acid saturation determines the fluidity and stability of plasma membrane, comprising important factors that influence its functional and regulatory properties.

Several studies have demonstrated that fatty acid synthesis and oxidation processes are altered during senescence [12, 13, 16]. These changes can be assessed using various techniques, including lipidomics [13, 17, 18].

FAs are not only essential constituents of membrane lipids, but also substrates for energy metabolism.

Fatty acid synthase (FASN) and acetyl‐CoA carboxylase are key rate‐limiting enzymes for fatty acid synthesis, and their expression is controlled by common lipogenic transcription factors [19].

FASN is a multifunctional enzyme that uses acetyl‐CoA and malonyl‐CoA as substrates and NADPH as reducing cofactor to obtain predominantly palmitate (C16). Under normal conditions, it converts carbohydrates into fatty acids, which are then stored as triacylglycerols, which are used later to obtain energy by β‐oxidation.

During tumorigenesis, the over‐proliferating cell population requires increased fatty acid biosynthesis for membranogenesis and lipid based post‐translational modifications. Indeed, FASN has been found to be present at high levels in many varieties of common human tumors [20]. Another enzyme involved in lipogenesis is the stearoyl‐CoA desaturase‐1 (SCD1). This enzyme, also called delta‐9 desaturase (Δ9), catalyzes the conversion of saturated fatty acyl‐CoAs, such as palmitic acid and stearic acid, into palmitoleic and oleic acid, respectively. Δ9 is the major isoform of human stearoyl‐CoA desaturases [21]. This is the rate limiting enzyme in the biosynthesis of MUFAs, which are then used as main substrates for the synthesis of various types of lipids, such as phospholipids, triglycerides and cholesterol esters. The SFA/MUFA ratio is one of the factors influencing the physical properties of the membrane, such as fluidity [22]. Elevated levels of Δ9 have been reported in several human tumors [23]. The development of a neoplastic phenotype correlates with a global change in the metabolism of MUFAs that could be selectively advantageous in cell growth and survival [24].

In cellular senescence, along with changes in lipid composition of cell membranes, specific variations in the levels of enzymes involved in lipid metabolism have also been observed. Experimental studies identified that fatty acid synthesis decreases during senescence because of a decline in the levels of the enzymes involved in fatty acid biosynthesis, such as FASN and Δ9 [19, 25]. On the other hand, the activity of phospholipases, which catalyze the hydrolysis of phospholipids, is usually upregulated in senescent cells and their expression has been demonstrated to promote premature senescence [12, 26].

Interestingly, senescence has been shown to be protective and beneficial for the cell, contributing to tissue repair and the protection against oncogenic factors [27, 28]. Changes in the lipidome are considered one of the cellular mechanisms capable of protecting senescent cells from lipotoxicity induced cellular damage as a result of their high oxidative stress environment [29].

Currently, the correct identification of senescent cells *in vitro* is not always clear because senescent cells often share some molecular and cellular features with cells undergoing differentiation and quiescence [30, 31]. Therefore, changes in cell morphology and markers based on the detection of proliferation or cell cycle arrest are not specific with respect to identifying senescent cells. In this regard, a more specific biomarker of senescence is the overexpression, at specific value of pH, of lysosomal beta‐galactosidase, which is not expressed by quiescent and differentiated cells [31].

Although the senescence‐associated beta‐galactosidase (SA‐β‐gal) assay can be considered a quick assessment of senescence, different assays and markers are recommended for identifying and defining the senescent cells. It is known that cancer cells avoid the replicative senescence, and so the senescence process must be induced either with a drug, genetic manipulation or stressor agent [32, 33]. In the present study, we propose a model of induced senescence in cancer cell lines to investigate cellular senescence through a lipidomic approach.

## Materials and methods

### Cell lines and culture media

Pancreatic cancer cell line (MIA‐PaCa‐2 and PANC‐1) and human hepatoma cells (Hepa‐RG) were purchased from American Tissue Culture Collection (ATCC, Manassas, VA, USA). Human hepatocellular carcinoma cells (HLF) were purchased from JCRB Cell Bank (Osaka, Japan). MIA‐PaCa‐2, PANC‐1 and HLF cells were cultured in Dulbecco's modified Eagle's medium (Thermo Fisher Scientific, Milan, Italy), whereas Hepa‐RG were cultured in hepatocyte bullet kit medium (Thermo Fisher Scientific) each of which was supplemented with 10% heat‐inactivated fetal bovine serum and 1% of Antibiotic‐Antimycotic (10 000 units·mL^−1^ penicillin, 10 000 μg·mL^−1^ streptomycin and 25 μg·mL^−1^ Gibco Amphotericin B penicillin). Cells were routinely propagated and cultured in a cell culture incubator at 37 °C and 5% CO_2_.

### Induction of *in‐vitro* cellular senescence

Cells were expanded in six‐well culture plate until the desired confluence was obtained (about 60%), then the culture medium was removed and replaced with medium added with doxorubicin (DOXO) at a final concentration of 50 nm. After 2–3 days of treatment, depending on the cell line, the medium was removed and replaced with fresh medium. After 5 days, the cells were appropriately lysed to perform the lipidomic analysis, SA‐β‐Gal assay and western blotting. The control sample comprised untreated cells.

To assess the correct induction of senescence, the SA‐β‐gal activity assay kit (Fluorescence, Plate‐Based; Cell Signaling Technology, Beverly, MA, USA) was used. Briefly, a 1× senescence cell lysis buffer was prepared on ice with the appropriate amount of phenylmethanesulfonyl fluoride protease inhibitor (1 mm) and protease/phosphatase inhibitor cocktail (100×). After aspirating the culture medium and washing the cells with phosphate‐buffered saline 1×, 500 μL of cold 1× senescence cell lysis buffer was added to each well of the six‐well culture plate and incubated on ice for 5 min.

The cells were scraped from the plate surface and lysed; the lysate was transferred into microcentrifuge tubes and centrifuged at 11 000 *g* for 5 min at 4 °C. The supernatant, containing the cell lysate, was transferred into new microcentrifuge tubes.

Next, in the dark, 2× assay buffer was prepared by adding 2× senescence reaction buffer, β‐mercaptoethanol at a final concentration of 10 mm and SA‐β‐gal substrate (20×) at a final con‐centration of 1×. Then, 50 μL of the cell lysate was added to a 96‐well plate with 50 μL of 2× assay buffer; the samples were incubated at 37 °C, protected from light, for 2 h. Subsequently, 50 μL of the reaction was transferred to a black opaque 96‐well plate with 200 μL of senescence stop solution for each well. Finally, fluorescence was read with a fluorescence plate reader set with excitation at 360 nm and emission at 465 nm. Fluorescence values were normalized to the total protein content of each sample. Total protein concentration was determined by the standard Bradford assay (Bio‐Rad Laboratories, Hercules, CA, USA).

### Fatty acid analysis

Senescent inducted and control cells were washed with phosphate‐buffered saline 1×, the supernatant was discarded and the cell pellet was used for lipidomic analysis.

Cellular lipids were extracted using a modified Bligh and Dyer method with chloroform/methanol/water (2:1:1.8; v/v/v) [34]. In particular, 0.450 mL of acidic saline solution and 2.250 mL of chloroform–methanol were added to the cell lysate. This solution was centrifuged at 2400 *g* for 20 min at 4 °C. The lower layer containing chloroform was taken and evaporated in an evaporator (Thermo Fisher Scientific).

Transesterification was performed with a mixture of toluene and boron trifluoride‐methanol solution (14% in methanol) in termobloch at 80 °C for 2 h. The resulting fatty acid methyl esters (FAMEs) were extracted in 0.500 mL of toluene and analyzed by GC.

The FAME obtained from cellular samples was separated by GC (Thermo Fisher Scientific) equipped with a splitless inlet, a flame ionization detector and a hydrogen gas generator (Thermo Fisher Scientific). In total, 1 μL of FAME was analyzed on a BPX70 0.25 UM capillary column (SGE Analytical Science, Milton Keynes, UK). Hydrogen was used as carrier gas (3 mL·min^−1^, constant flow mode). The temperature of the injector and the flame ionization detector was 250 °C. FA quantification was expressed as relative percentages of total FA content. Peaks were identified by comparing them with a mixture of standards (Supelco 37‐Component FAME Mix; Sigma‐Aldrich, Milan, Italy).

Among the 37 FAs analyzed in the cell membranes, specific attention was given to key representatives of the two main FA families: SFAs, especially palmitic and stearic acid, and MUFAs, such as palmitoleic and oleic acid. In particular, Δ9 was calculated as the sum of the ratios palmitoleic/palmitic acid and oleic/stearic acid, representing the activities of the two desaturase enzymes.

### Protein extraction and western blot analysis

Protein extraction and western blot analysis were performed on senescence inducted and untreated cells. Total proteins from cell lines were extracted using a volume of five times the volume of the pellet of RIPA buffer (Sigma‐Aldrich) with added Halt Protease & Phosphatase Inhibitor (Thermo Fisher Scientific). The protein concentration was evaluated by a standard Bradford assay (Bio‐Rad Laboratories). Next, 50 μg of proteins from each sample was separated via 4–15% Tris‐glycine SDS/PAGE (Bio‐Rad Laboratories). Membranes were incubated with the following antibodies: Δ9 (SCD1; dilution 1:400; Cell Signaling Technology), FASN (dilution 1:500; Immunological Sciences), p21 (dilution 1:500; Cell Signaling), SASP Ab Sempler kit‐ 38 461 T (PAI‐1; dilution 1:400; and IL‐6; dilution 1:400; Cell Signaling Technology) and β‐actin (dilution 1:1000; Cell Signaling Technology).

After overnight incubation, an anti‐rabbit secondary antibody (dilution 1:3000; Bio‐Rad Laboratories) was used. The chemiluminescence signal from proteins was revealed using Clarity Western ECL Substrate or Clarity Max Western ECL Substrate (Bio‐Rad Laboratories) and analyzed using a chemiluminescence detection system ChemiDoc XRS (Bio‐Rad Laboratories). Experiments were conducted in triplicate. The relative density of the bands was calculated using image lab, version 5.2.1 (Bio‐Rad Laboratories) and the proteins detected were normalized against β‐actin signal. Bar graphs were generated using prism, version 8 (GraphPad Software Inc., San Diego, CA, USA).

### Statistical analysis

An unpaired *t*‐test with Welch's correction was performed to test differences in protein expression between control cells (CTR) and cells treated with DOXO. Ordinary one‐way analysis of variance (ANOVA) corrected for multiple comparison by Bonferroni's post‐hoc analysis was performed to compare differences in lipidomic profiles. *P* ≤ 0.003 was considered statistically significant.

## Results

**Fig. 1:**
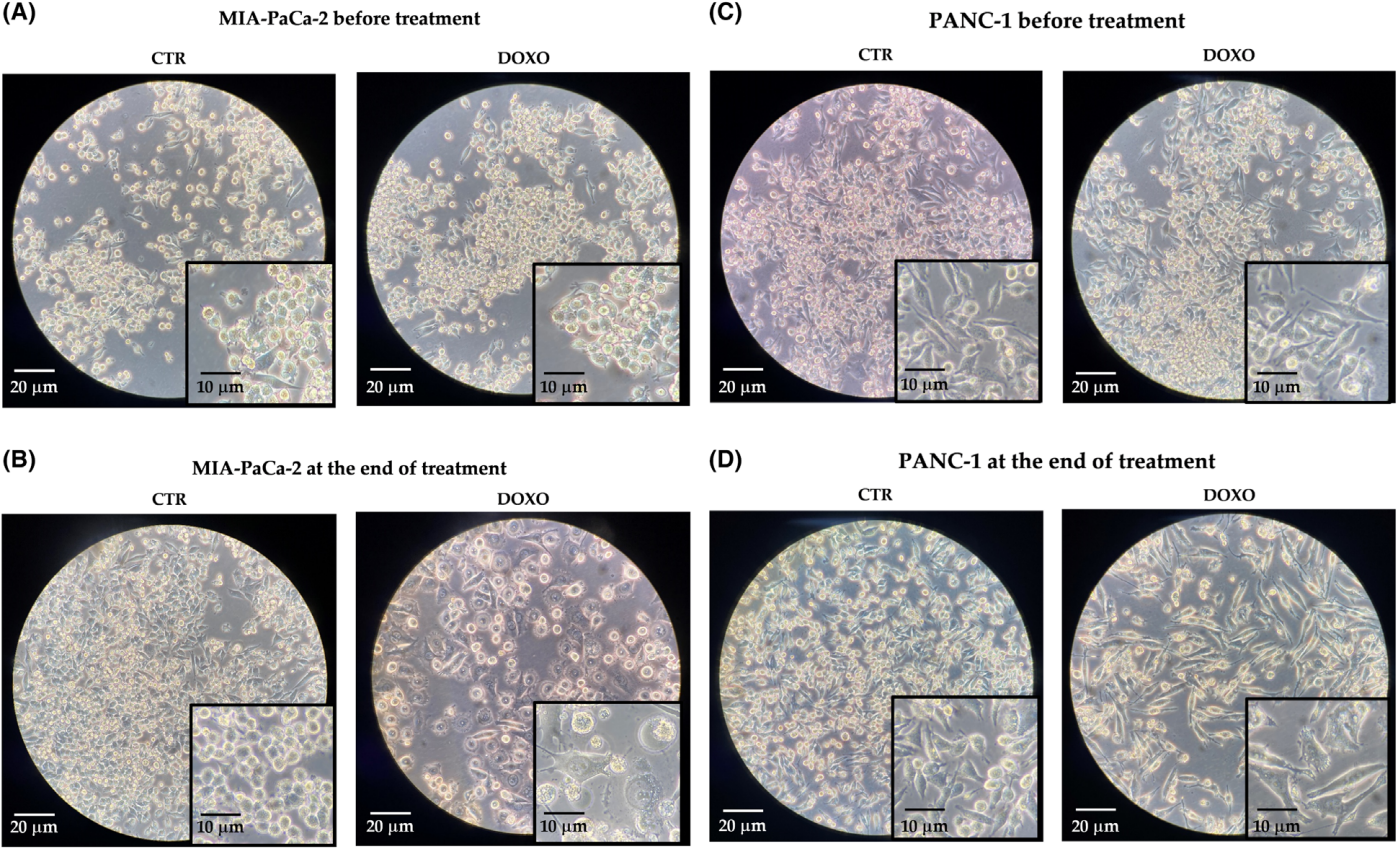
Size and morphology in two different magnifications, 20× (scale bar = 20 μm) and 40× (scale bar = 10 μm) of MIA‐PaCa‐2 cells untreated (CTR) and treated with 50 μm doxorubicin (DOXO) before (A) and after treatment (B). Size and morphology in two different magnifications, 20× (scale bar = 20 μm) and 40× (scale bar = 10 μm) of PANC‐1 cells untreated (CTR) and treated with 50 μm doxorubicin (DOXO) before (C) and after treatment (D).

**Fig. 2:**
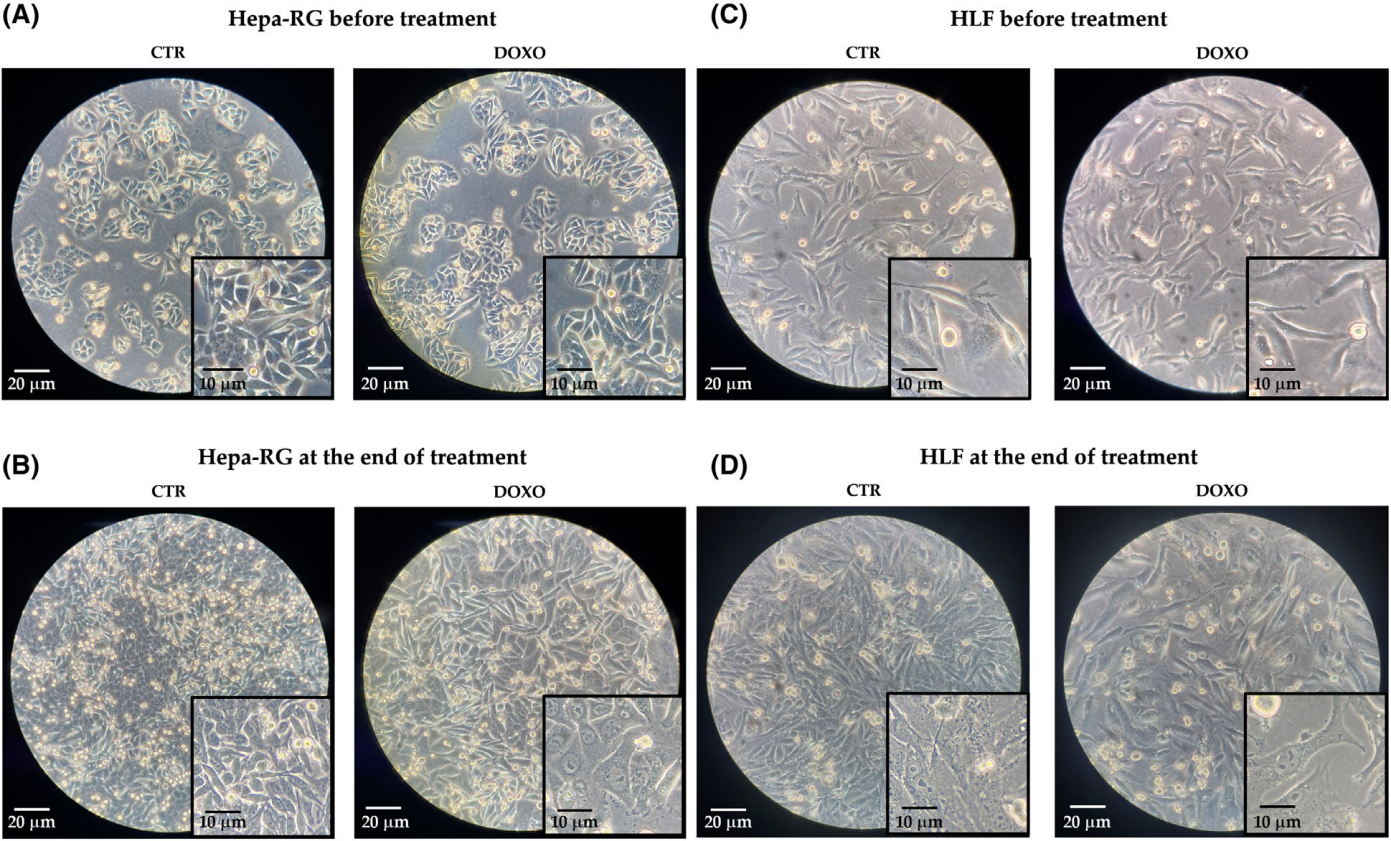
Size and morphology in two different magnifications, 20× (scale bar = 20 μm) and 40× (scale bar = 10 μm) of Hepa‐RG cells untreated (CTR) and treated with 50 μm doxorubicin (DOXO) before (A) and after treatment (B). Size and morphology in two different magnifications, 20× (scale bar = 20 μm) and 40× (scale bar = 10 μm) of HLF cells untreated (CTR) and treated with 50 μm doxorubicin (DOXO) before (C) and after treatment (D).

After treatment with DOXO (50 nm), all of the cell lines used (MIA‐PaCa‐2, PANC‐1, Hepa‐RG and HLF) showed changes, as evaluated by microscopic visualization, in their size and morphology (Figs 1 and 2). Figure 1 shows the morphology in MIA‐PaCa‐2 (Fig. 1A,B) and PANC‐1 (Fig. 1C,D) before and at the end of treatment, whereas the morphology in Hepa‐RG and HLF is shown in Fig. 2A–D, respectively. At the end of treatment, the cells exhibit an enlarged cytoplasmic compartment and flattened morphology, in association with slowed growth. To confirm that the treated cell lines had achieved senescence, the presence of SA‐β‐gal (i.e. as a marker of senescence) was determined [35, 36].

**Fig. 3:**
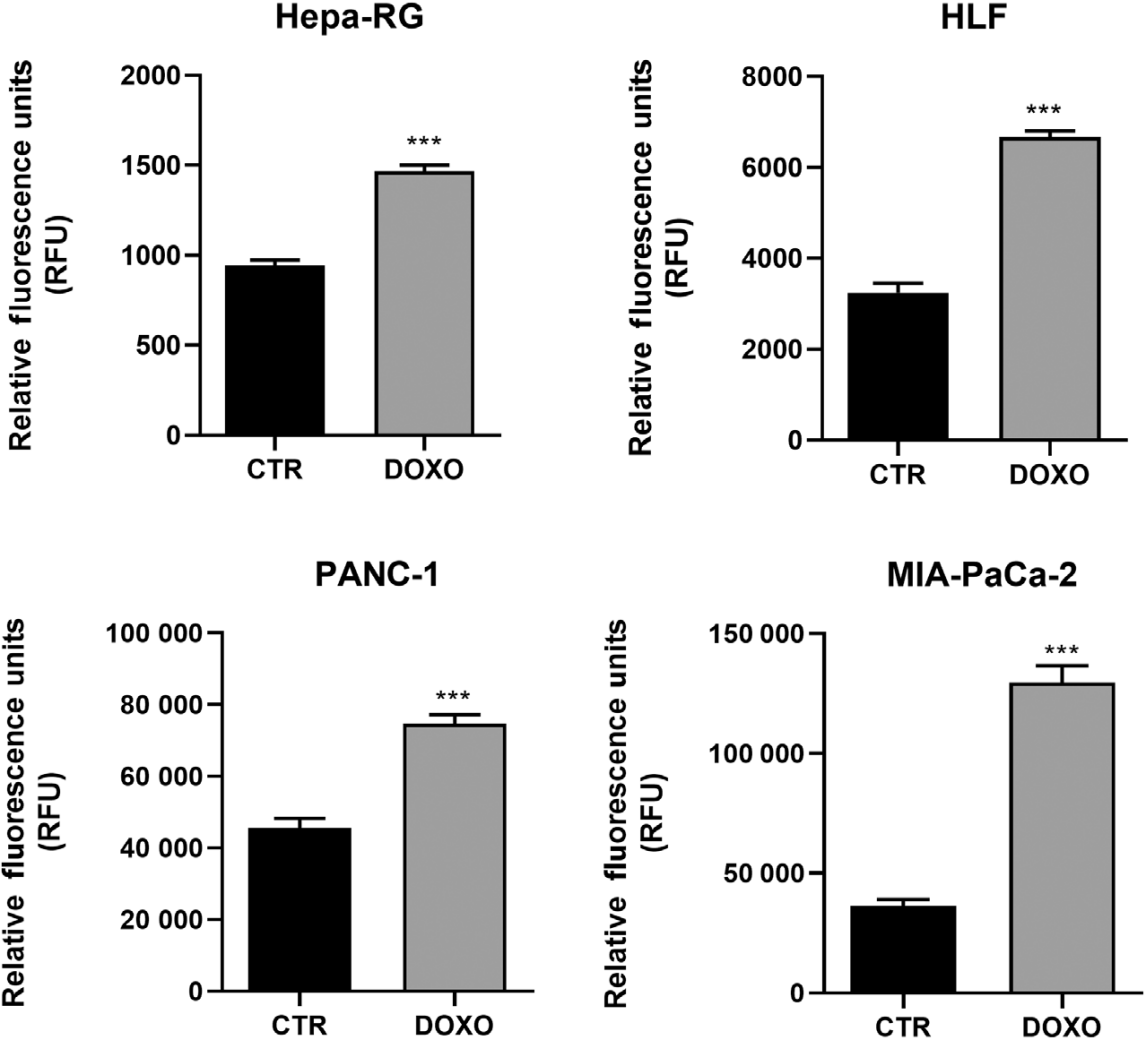
Senescence‐associated β‐galactosidase (SA‐β‐gal) activity expressed in relative fluorescence units (RFU) of untreated control (CTR) and treated with doxorubicin 50 nm (DOXO) in MIA‐PaCa‐2 and PANC‐1, Hepa‐RG and HLF cell lines. All data represent the results of nine biologically‐independent replicates, expressed as the mean ± SD. ***P ≤ 0.0002 indicates statistically significant differences by an unpaired *t*‐test with Welch's correction.

Figure 3 shows the results of the SA‐β‐gal activity assay, which was significantly increased after treatment with 50 nm DOXO in all cells tested.

**Fig. 4:**
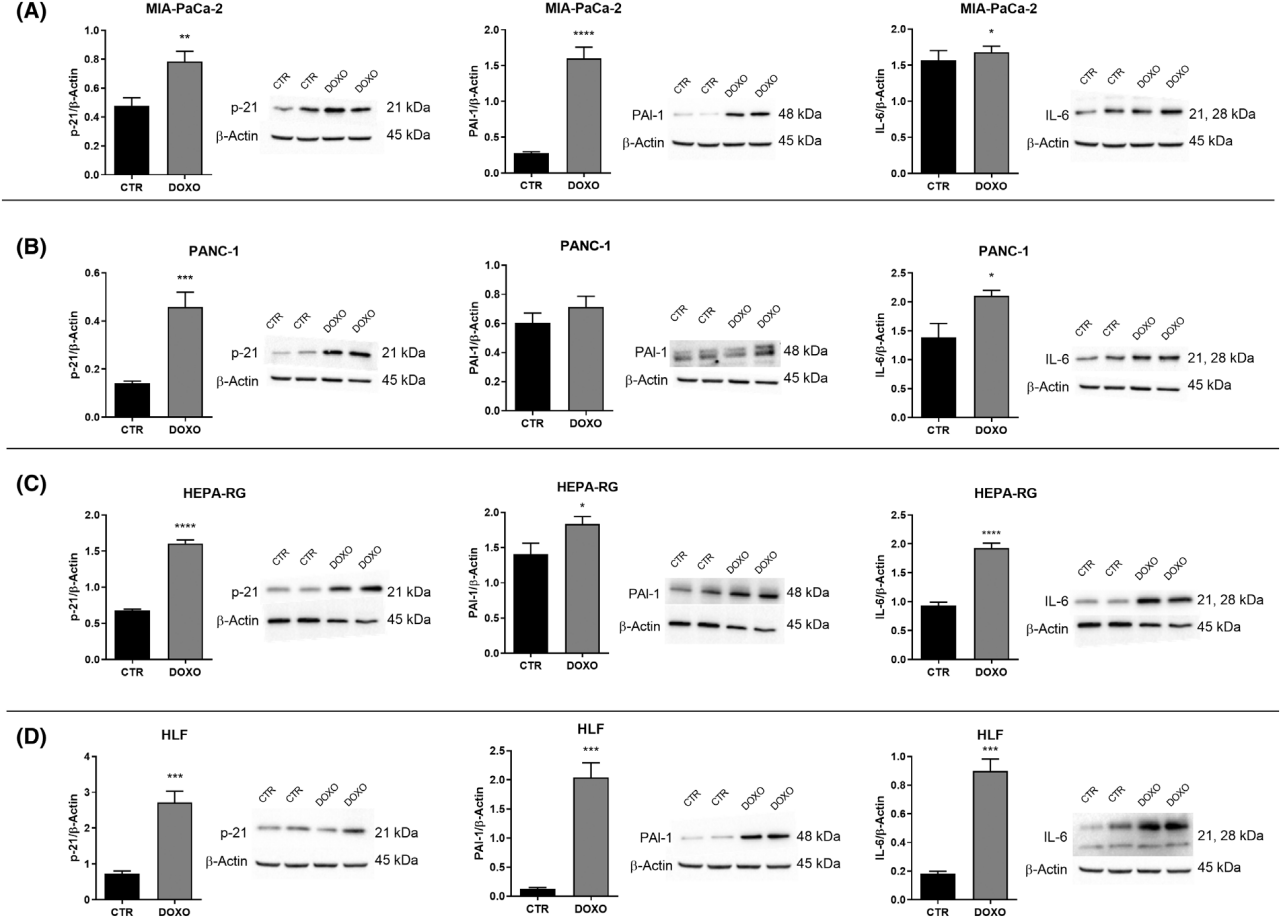
p21, PAI‐1, and IL‐6 protein expression levels and representative western blot bands detected in untreated control cells (CTR) and treated with doxorubicin 50 nm (DOXO) in (A) MIA‐PaCa‐2, (B) PANC‐1, (C) Hepa‐RG and (D) HLF cells. All data represent the results of nine biologically‐independent replicates, expressed as the mean ± SD. *P ≤ 0.003, **P ≤ 0.002, ***P ≤ 0.0002 and ****P ≤ 0.0001 indicate statistically significant differences by an unpaired *t*‐test with Welch's correction.

These results were confirmed by western blot analysis of the protein levels of p21, as involved in regulation of cell cycle, and of plasminogen activator inhibitor‐1 (PAI‐1) and interleukin‐6 (IL‐6), comprising two markers of senescence and elements of SASP, Figure 4 shows higher protein expression levels of p21, PAI‐1 and IL‐6 in cells treated with DOXO than in CTR cells. The expression level of each investigated protein was normalized with the expression level of β‐actin relative to each of the cell lines.

To examine the changes in the lipid composition of cell membranes, lipidomic analysis was performed in the cell lines treated with 50 nm DOXO and in the control cells.

**Fig. 5:**
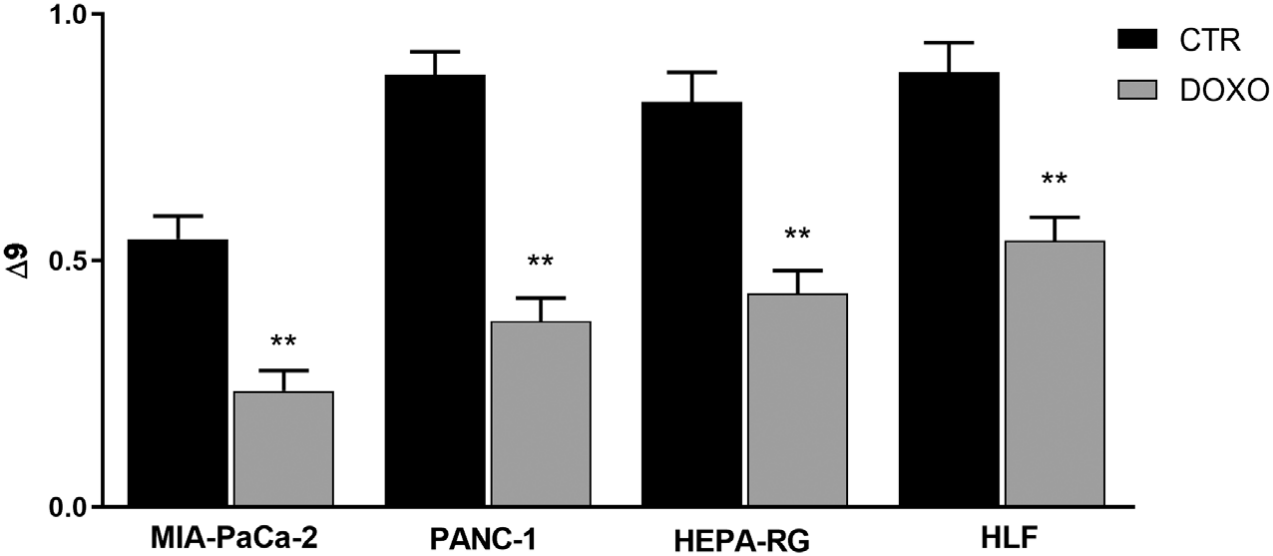
Δ9 values in membrane fatty acids from untreated control (CTR) and doxorubicin‐treated (DOXO) MIA‐PaCa‐2, PANC‐1, Hepa‐RG and HLF cells. All data represent the results of nine biologically‐independent samples expressed as the mean ± SD. **P ≤ 0.002 indicate statistically significant differences by ordinary one‐way ANOVA corrected for multiple comparison by Bonferroni's post‐hoc analysis.

**Fig. 6:**
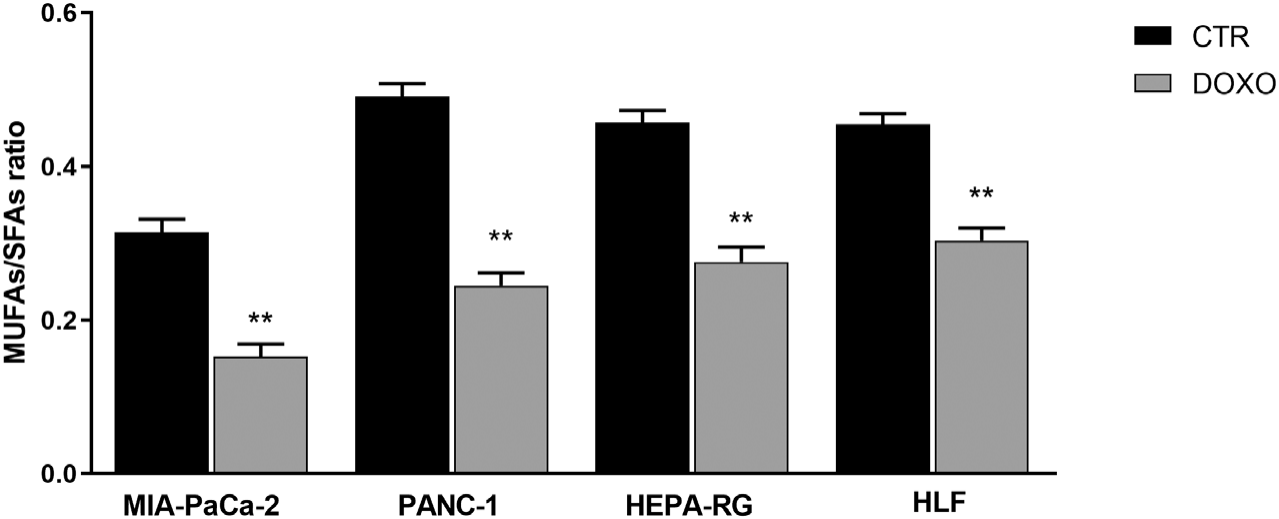
MUFAs/SFAs ratio in membrane fatty acids from untreated control (CTR) and doxorubicin‐treated (DOXO) MIA‐PaCa‐2, PANC‐1, Hepa‐RG and HLF cells. All data represent the results of nine biologically‐independent samples expressed as the mean ± SD. **P ≤ 0.002 indicates statistically significant differences by ordinary one‐way ANOVA corrected for multiple comparison by Bonferroni's post‐hoc analysis.

Table 1 shows the relative percentage of membrane fatty acids in MIA‐PaCa‐2 and PANC‐1 cell lines before and after treatment. Interestingly, in the DOXO treated cells, there was a significant increase in SFAs, such as palmitic and stearic fatty acid, compared to CTR cells. By contrast, there was a reduction in oleic and palmitoleic acid content, associated with a statistically significant decrease in the levels of MUFAs. Consequently, Δ9, which is given by the sum of the ratios palmitoleic/palmitic acid and oleic/stearic acid, representing the activities of the two desaturase enzymes, was significantly reduced in DOXO treated cells compared to CTR cells (Fig. 5), and the MUFA/SFA ratio was decreased in senescent cells (Fig. 6), comprising an indicator of Δ9 enzyme activity.

**Table 1:** Relative percentage of membrane fatty acids profile in MIA‐PaCa‐2 and PANC‐1 cell lines untreated (CTR) and treated with doxorubicin 50 nm (DOXO). All values are expressed as the mean ± SD of nine biologically‐independent samples. *P < 0.03 and **P < 0.002 by ordinary one‐way ANOVA corrected for multiple comparison by Bonferroni's post‐hoc analysis. MUFA, monounsaturated fatty acid; SFA, saturated fatty acid. ns, not significant.

%	MIA‐PaCa‐2			PANC‐1		
	CTR	DOXO	*P*	CTR	DOXO	*P*
Fatty acid profile						
Palmitic acid	33.07 ± 4.54	45.30 ± 4.67*		34.40 ± 4.45	41.12 ± 4.50*	
Palmitoleic acid	1.38 ± 0.48	0.67 ± 0.46*		1.54 ± 0.52	0.97 ± 0.47 ns	
Stearic acid	23.10 ± 4.47	30.78 ± 4.58*		18.18 ± 3.41	25.12 ± 3.02**	
Oleic acid	14.05 ± 3.39	8.90 ± 2.83 ns		15.87 ± 3.39	11.90 ± 3.39 ns	
Total fatty acids						
Palmitoleic acid/palmitc acid	0.04 ± 0.01	0.01 ± 0.01*		0.04 ± 0.01	0.02 ± 0.01*	
Oleic acid/stearic acid	0.62 ± 0.15	0.29 ± 0.07*		0.87 ± 0.06	0.48 ± 0.16**	
SFAs	63.79 ± 4.50	78.22 ± 4.79**		55.67 ± 4.43	69.76 ± 4.47**	
MUFAs	20.29 ± 4.56	12.08 ± 4.53*		27.53 ± 4.72	17.36 ± 4.60**	

The same behavior was observed in the Hepa‐RG and HLF cell lines. Table 2 shows an increase in the relative percentage of palmitoleic and stearic acid and a decrease in palmitoleic and oleic acid in the DOXO treated cells compared to CTR cells. Consequently, a significant reduction in Δ9 was detected (Fig. 5). Even the total fatty acids showed the same behavior; indeed, in Hepa‐RG and HLF cells, there was a significant increase in SFAs and a decrease in MUFAs and the MUFA/SFA ratio (Fig. 6).

**Table 2:** Relative percentage of membrane fatty acids profile in Hepa‐RG and HLF cell lines untreated (CTR) and treated with doxorubicin 50 nm (DOXO). All values are expressed as the mean ± SD of nine biologically‐independent samples. *P < 0.03 and **P < 0.002 by ordinary one‐way ANOVA corrected for multiple comparison by Bonferroni's post‐hoc analysis. MUFA, monounsaturated fatty acid; SFA, saturated fatty acid. ns, not significant.

%	Hepa‐RG			HLF		
	CTR	DOXO	*P*	CTR	DOXO	*P*
Fatty acid profile						
Palmitic acid	29.35 ± 4.97	35.85 ± 4.43*		34.95 ± 4.51	39.62 ± 4.57 ns	
Palmitoleic acid	1.13 ± 0.46	0.75 ± 0.46 ns		1.57 ± 0.48	1.53 ± 0.47 ns	
Stearic acid	18.79 ± 3.06	23.28 ± 2.95*		18.80 ± 2.95	21.51 ± 2.93 ns	
Oleic acid	16.70 ± 3.50	12.61 ± 3.49 ns		17.76 ± 3.40	13.71 ± 3.41 ns	
Total fatty acids						
Palmitoleic acid/palmitc acid	0.04 ± 0.01	0.02 ± 0.01*		0.04 ± 0.01	0.04 ± 0.01 ns	
Oleic acid/stearic acid	0.92 ± 0.28	0.55 ± 0.18**		0.97 ± 0.28	0.65 ± 0.20*	
SFAs	51.76 ± 4.92	62.01 ± 4.43**		57.66 ± 4.52	65.63 ± 3.58*	
MUFAs	23.87 ± 4.72	17.33 ± 4.60*		26.36 ± 4.55	19.94 ± 4.48*	

**Fig. 7:**
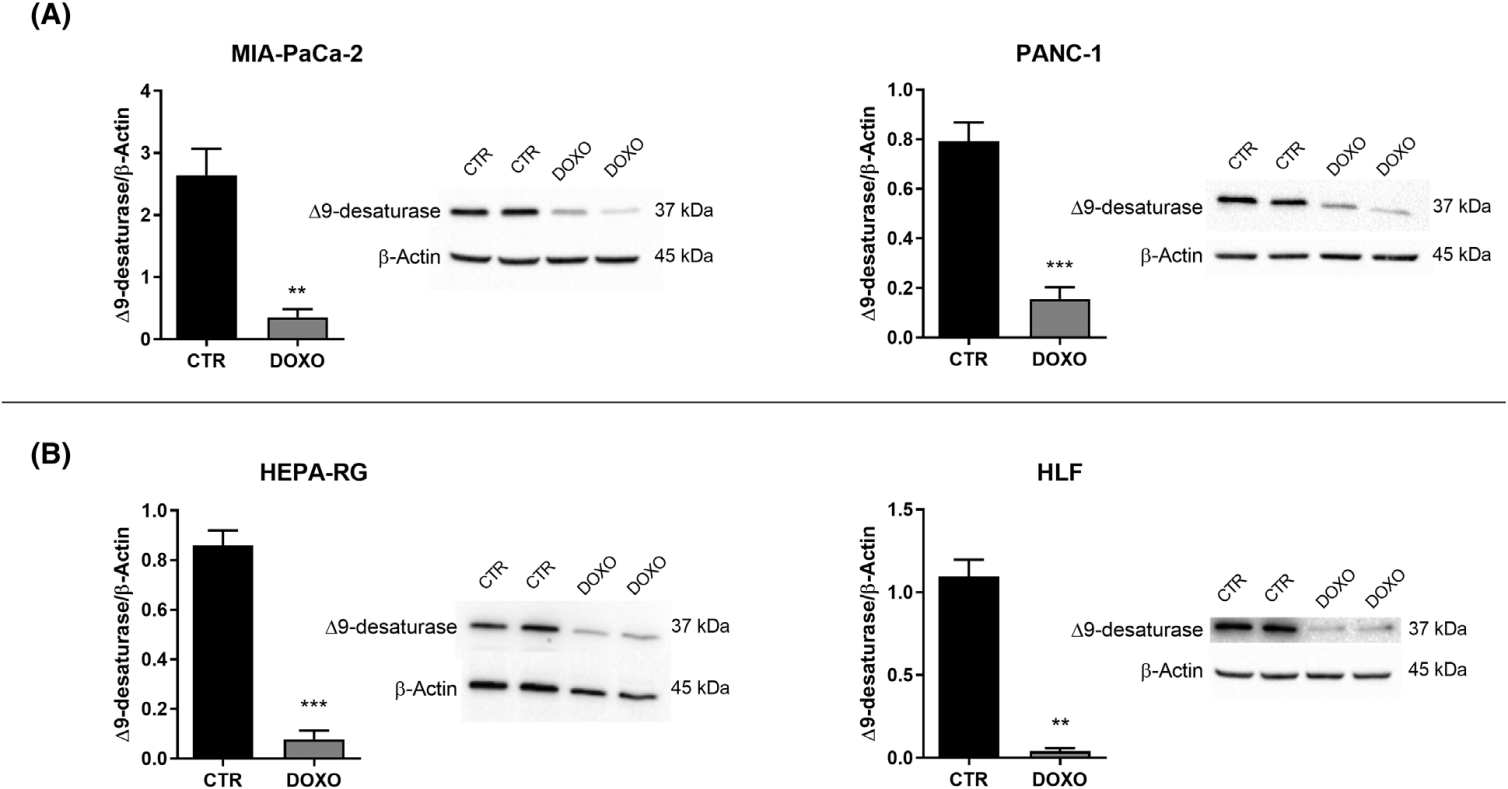
Delta‐9 desaturase (Δ9) protein expression level and representative western blot bands detected in untreated control (CTR) doxorubicin‐treated (DOXO) MIA‐PaCa‐2, PANC‐1 cells (A), Hepa‐RG and HLF cells (B). All data represent the results of nine biologically‐independent replicates, expressed as the mean ± SD. **P ≤ 0.002 and ***P ≤ 0.0002 indicate statistically significant differences by an unpaired *t*‐test with Welch's correction.

**Fig. 8:**
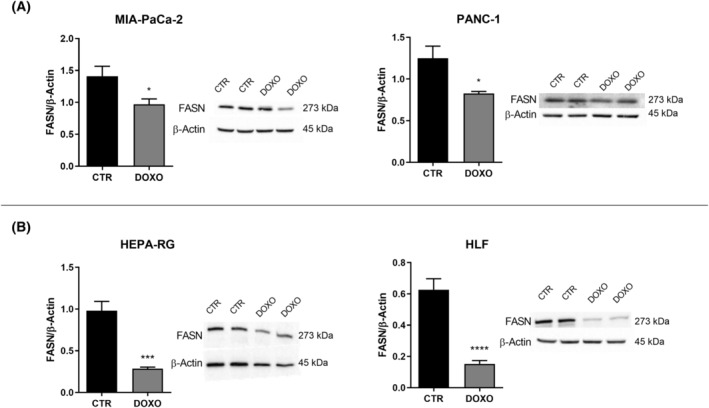
Fatty acid synthase (FASN) protein expression level and representative western blot bands detected in untreated control (CTR) doxorubicin‐treated (DOXO) MIA‐PaCa‐2, PANC‐1 cells (A), Hepa‐RG and HLF cells (B). All data represent the results of nine biologically‐independent replicates, expressed as the mean ± SD. *P ≤ 0.003, ***P ≤ 0.0002 and ****P ≤ 0.0001 indicate statistically significant differences by an unpaired *t*‐test with Welch's correction.

The reduction of Δ9 in senescent cells was also confirmed by western blot analysis. As shown in Fig. 7, a significant decrease in Δ9 protein level was demonstrated in the senescent MIA‐PaCa‐2, PANC‐1, Hepa‐RG and HLF cell lines compared to the CTR. Furthermore, a significant reduction of FASN expression in the senescent cells could be observed (Fig. 8).

## Discussion

Cellular senescence, both induced or replicative, is a specialized form of growth arrest, usually irreversible [1, 2, 3, 4, 5, 6, 7, 8, 9, 10, 11, 12, 13, 14, 15, 16, 17, 18, 19, 20, 21, 22, 23, 24, 25, 26, 27, 28, 29, 30, 31, 32, 33, 34, 35, 36, 37]. Senescence induction is a complex process and it is characterized by a series of molecular changes, involving lipid metabolism and cellular lipid content [12, 13]. The major metabolic processes involved in cellular senescence include uptake, transport, synthesis and oxidation, which are closely associated with aging and the process of SASP secretion [13].

In the present study, we analyzed the expression levels of Δ9, an enzyme involved in lipid metabolism, in different cell lines from pancreatic cancer (MIA‐PaCa‐2, PANC‐1) and liver (Hepa‐RG, HLF), induced to senescence by the chemotherapy drug doxorubicin. This drug acts by intercalating the DNA and preventing topoisomerase II from joining the rupture of the double strand of DNA [36].

In all cell lines used, a significant reduction in the levels of this enzyme has been demonstrated. This evidence suggests a correlation between Δ9 expression levels and the senescent cellular phenotype.

The present study provides the first evidence of a reduction in Δ9 levels as assessed through a lipidomic approach, using chromatographic analysis of lipids belonging to senescent cells and untreated control cells.

The correct induction of senescence was initially confirmed with the SA‐β‐Gal assay, showing the increase of lysosomal beta‐galactosidase in senescent cells compared to control cells.

To confirm the induction of senescence in the cells, we evaluated the expression levels of p21, and two components of SASP, such as interleukin‐6 (IL‐6) and plasminogen activator inhibitor‐1 (PAI‐1). The increase of p21, a cell cycle regulator, leads to persistent activation of RB family proteins, inhibition of E2F transcription factor transactivation, and subsequent cell cycle arrest [38].

Protein expression of pro‐inflammatory IL‐6 and PAI‐1, components of SASP, was higher in senescent cells than in control cells.

The cellular senescent condition is also characterized by changes in lipid metabolism. Senescent cells generally exhibit an altered cellular lipid profile as a result of impaired lipid metabolic pathways, although the nature of the changes in specific lipid subsets appears to be dependent on cell type [18].

The membrane content of SFAs can be considered as a new biomarker of cellular senescence, known to play a debilitating role in age‐related diseases. In particular, an excess of SFAs is damaging to cells. We focused our attention on Δ9, a desaturase that converts the SFAs palmitic and stearic acid into their monounsaturated forms, palmitoleic and oleic acid [39]. The regulation of Δ9 activity is of great physiological and pathological importance because most lipid functions, such as membrane fluidity, lipoprotein metabolism and energy storage, are dependent on it.

High levels of Δ9, and consequently MUFAs, have been positively correlated with the aggressiveness and malignancy of numerous tumors. By contrast, a decrease in Δ9 can also contribute to the beginning of the process of cellular senescence, whereas the inhibition of Δ9 activity leads to the death of cancer cell as a result of the depletion of MUFA, as demonstrated previously [40, 41, 42].

Data analysis of Δ9 protein expression in all four cell lines, as evaluated by western blot analysis, agrees with the lipidomic analysis, and underlines the decrease of Δ9 protein levels in senescent cells.

Previously, we demonstrated the ability of two table grape varieties to inhibit Δ9 in two human colorectal cancer cell lines with different degrees of differentiation, blocking cell migration and affecting the membrane fluidity [43]. This shows how Δ9 certainly plays an important role in the adaptation of cancer cells through the mechanism of cellular senescence.

The down regulation of FASN observed in the senescence‐induced cells could indicate a mechanism of regulation of lipogenesis that results in the inhibition of cell replication.

Overexpression and increased FASN activity have indeed been observed in cancer cells, suggesting that fatty acids can be utilized for cell replication [44]. Instead, senescent cells lose the replicative capacity and so there is no need to produce these metabolites, such that down‐regulation of the FASN can be observed.

It has also been reported that *de novo* synthesis of fatty acids and their conversion to MUFAs are necessary for cell proliferation. Accordingly, in senescent cells, the down‐regulation of *de novo* fatty acid synthesis and the desaturation could suggest a lipogenesis regulation mechanism associated with the irreversible arrest of the cell replication process.

Various proteomic and lipidomic approaches are used in different human cells to investigate the activity of metabolic enzymes and cellular components involved in cell growth and proliferation.

Therefore, the study of fatty acids in the membrane phospholipid bilayer comprises a non‐invasive way of assessing the behavior of cells during their life cycle.

In conclusion, we have demonstrated that the study of cell membrane composition could potentially form the basis for future applications investigating the state of cellular senescence and we propose that Δ9 enzyme content is a biomarker of this cell phenotype.

### Limitations of the study

Although it is clear that there is an association between Δ9 down‐regulation and cell senescence in gastrointestinal cells, further experimental studies will be needed to determine whether Δ9 modulation also occurs in other types of cell lines induced to senescence.

## Conflicts of interest

The authors declare that they have no conflicts of interest.

## Author contributions

MN conceived and designed the project. VDN, EAC, GP, MiCo and IS performed the analyses. VDN and EAC edited the data. VDN, EAC and MN prepared the original draft. MaCe and MN viewed the original draft. MN reviewed and modified the original draft. All authors have read and approved the final version of the manuscript submitted for publication.

## Data Availability

Data are available from the corresponding author upon reasonable request.
